# Evaluation and Analysis of Absence of Homozygosity (AOH) Using Chromosome Analysis by Medium Coverage Whole Genome Sequencing (CMA-seq) in Prenatal Diagnosis

**DOI:** 10.3390/diagnostics13030560

**Published:** 2023-02-02

**Authors:** Yan Lü, Yulin Jiang, Xiya Zhou, Na Hao, Guizhen Lü, Xiangxue Guo, Ruidong Guo, Wenjie Liu, Chenlu Xu, Jiazhen Chang, Mengmeng Li, Hanzhe Zhang, Jing Zhou, Wei (Victor) Zhang, Qingwei Qi

**Affiliations:** 1 Department of Obstetrics and Gynecology, Peking Union Medical College Hospital, Peking Union Medical College & Chinese Academy of Medical Sciences, Beijing 100730, China; 2 AmCare Genomics Lab, Guangzhou 510335, China

**Keywords:** absence of homozygosity (AOH), medium coverage genome sequencing, chromosomal microarray analysis (CMA), prenatal diagnosis

## Abstract

Objective: Absence of homozygosity (AOH) is a genetic characteristic known to cause human diseases mainly through autosomal recessive or imprinting mechanisms. The importance and necessity of accurate AOH detection has become more clinically significant in recent years. However, it remains a challenging task for sequencing-based methods thus far. Methods: In this study, we developed and optimized a new bioinformatic algorithm based on the assessment of minimum sequencing coverage, optimal bin size, the Z-score threshold of four types of allele count and the frequency for accurate genotyping using 28 AOH negative samples, and redefined the AOH detection cutoff value. We showed the performance of chromosome analysis by five-fold coverage whole genome sequencing (CMA-seq) for AOH identification in 27 typical prenatal/postnatal AOH positive samples, which were previously confirmed by chromosomal microarray analysis with single nucleotide polymorphism array (CMA/SNP array). Results: The blinded study indicated that for all three forms of AOH, including whole genomic AOH, single chromosomal AOH and segmental AOH, and all kinds of sample types, including chorionic villus sampling, amniotic fluid, cord blood, peripheral blood and abortive tissue, CMA-seq showed equivalent detection power to that of routine CMA/SNP arrays (750K). The subtle difference between the two methods is that CMA-seq is prone to detect small inconsecutive AOHs, while CMA/SNP array reports it as a whole. Conclusion: Based on our newly developed bioinformatic algorithm, it is feasible to detect clinically significant AOH using CMA-seq in prenatal diagnosis.

## 1. Introduction

Chromosomal microarray analysis (CMA) has been initially recommended as a first-tier clinical genetic tool for the evaluation of postnatal patients with suspected developmental disabilities or congenital anomalies [1]. During the last decade, chromosomal microarray analysis with single nucleotide polymorphism arrays (CMA/SNP arrays) has enabled the rapid advancement of genome-wide detection of copy number variations (CNVs) at a relatively medium resolution for the discovery of microdeletion and microduplication syndromes [2]. More importantly, CMA/SNP arrays can also provide clinically useful information regarding the absence of homozygosity/uniparental disomy (AOH/UPD) in copy-number neutral scenarios and polyploidy by genotyping [3,4,5]. Therefore, CMA/SNP arrays are recommended for first-tier testing in the prenatal setting for fetuses with structural anomalies [3,6,7]. Since AOH is associated with autosomal recessive diseases and imprinting disorders resulting from UPD, the detection of AOH is of significant clinical importance in both prenatal and postnatal settings.

Recently population-level genomic studies using sequencing technologies have provided tremendous new insights regarding the clinical importance of AOH/UPD. First, the estimated prevalence of AOH/UPD based on the general population of approximately 4 million individuals revealed that its occurrence is 1 in 2000 births, which is a nearly 2× increase compared to previous estimations [8]. It is even more significantly higher (1 in 167) in a pediatric patient cohort with a broad spectrum of clinical manifestations subjected to exome sequencing [9]. Second, one of the plausible factors accounting for such a huge discrepancy in prevalence is the possibility that the hybridization-based microarray approach has an intrinsic resolution limitation, which directly impacts the detection sensitivity of AOH. The limitation arises mainly from the nonuniform probe distribution and the resulting nonuniform genomic coverage, which are known at the beginning of this technology [10]. A recent study showed that each individual carries, on average, 2.9 rare structural variants (SVs) affecting coding regions and 19.1 rare noncoding deletions. The mutation burden of CNVs is nearly equal to that of loss-of-function single nucleotide variants (SNVs) and indel variants, which is only just being realized by the scientific community [11]. Third, as the importance of detecting variants both from coding and noncoding regions is being realized in clinical tests, the guideline for clinical interpretation of variants found in all loci across whole genome regions has been recently developed and published [12].

The successful application of sequencing technologies has been exemplified by CNV detection in clinical settings. Low-pass whole-genome sequencing (LP-WGS) or CNV sequencing (CNV-seq) with ~0.1–1× coverage depth was reported for clinical application and widely used for CNV detection [13,14,15,16]. For example, Dong et al. used a ~0.25× coverage LP-WGS approach in CNV analysis to identify aneuploidies, pathogenic CNVs and chromosomal mosaicism as low as 25% [7,17]. Our group also reported the detection of CNVs for prenatal diagnosis with LP-WGS [18]. However, in most cases, additional testing is required for AOH detection, either short tandem repeat markers or methylation analyses.

The clinical importance and necessity of accurate AOH detection has become more significant, particularly for fetuses with abnormal ultrasound findings, while detection and clinical interpretation remain challenging tasks for the sequencing-based methods thus far. First, genotyping the B-allele by LP-WGS is difficult or infeasible via SNP information. Even with the tremendous advantages of LP-WGS compared with the CMA/SNP array for CNV detection, the inability to reliably detect AOH limits the sequencing-based approach for wide clinical application [19,20,21]. Second, only a few studies have investigated the feasibility of AOH detection by medium-pass genome sequencing [22,23], and the key bioinformatic algorithm has not yet been fully developed and optimized. Therefore, illustrating the detailed bioinformatic algorithm, as well as systematically evaluating its performance in clinical samples, is warranted.

In this study, we first performed sequencing at a series of different coverage depths and various genomic window sizes to demonstrate the minimal sequencing coverage depth for reliable AOH detection. We then used this method, called chromosome analysis by medium coverage whole genome sequencing (CMA-seq), in AOH detection, using different types of prenatal/postnatal samples previously confirmed by the CMA/SNP array. We demonstrated that SNVs within a 200 kb bin size window calling from a minimal 5X sequencing coverage yield sufficient information for accurate AOH detection, and the CMA-seq results are equivalent to those of the CMA/SNP array using our newly developed and optimized bioinformatic algorithms.

## 2. Materials and Methods

### 2.1. Study Design and Sample Preparation

**Figure 1:**
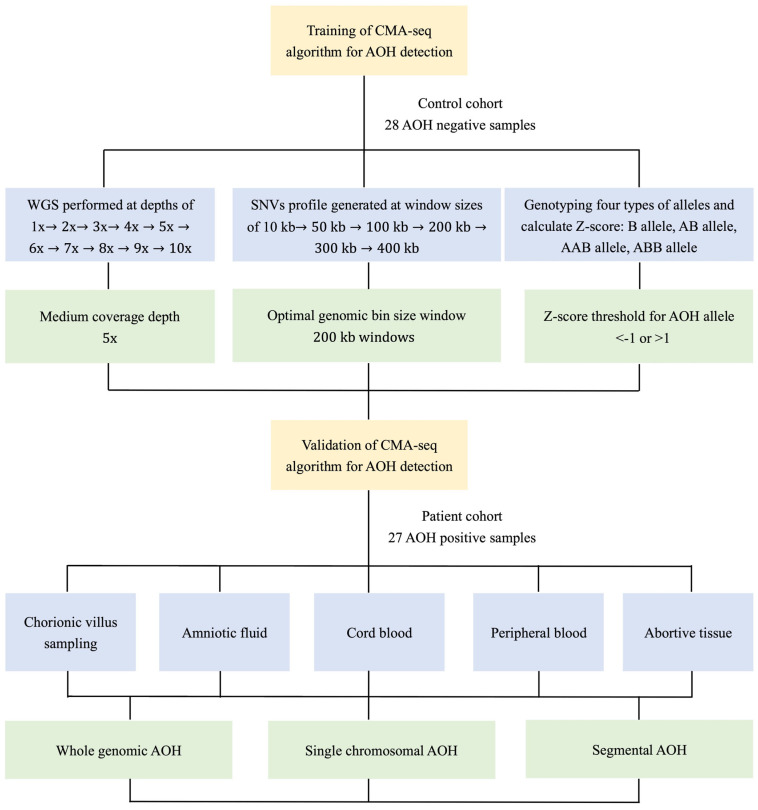
The flow chart of development and validation of CMA-seq algorithm for AOH detection.

The study protocol was approved by the medical ethics committee of Peking Union Medical College Hospital. The analyses of anonymized samples and reporting of deidentified molecular data with minimum clinical information were approved by the Institutional Review Board of Peking Union Medical College Hospital. In our study, twenty-eight samples with a negative AOH finding, verified by the CMA/SNP array (750k), were used as control samples for the training set of algorithm development. In the following validation stage of algorithm development, a total of 27 clinical samples were enrolled as the patient cohort, including prenatal/postnatal cases (chorionic villus sampling (CVS, 5), amniotic fluid (AF, 12), cord blood (CB, 1), peripheral blood (PB, 8) and abortive tissue (AB, 1)) with a positive finding of AOH by the CMA/SNP array (750K). The PB group consisted of adult samples with positive AOH findings that were clinically collected when the testing result of the fetal sample was abnormal and parental validation was warranted. For objectivity, detailed results of the AOH derived from CMA/SNP array were blinded across the whole process of CMA-seq testing and unclosed at the end of the CMA-seq experiment and analysis. Figure 1 illustrates the flow chart of development and validation of CMA-seq algorithm for AOH detection.

### 2.2. Whole Genome Sequencing at Different Coverage Depths

Genomic DNA from CVS and uncultured AF, CB, PB or AB was extracted with the standard operating instructions of the QIAamp DNA Blood Mini Kit (Qiagen, Valencia, CA, USA). Genomic DNA was broken by random fragmentation with an ultra sonicator Q800R (Qsonica, Newtown, CT, USA). Library construction and sequencing on the AmCareSeq-2000 sequencer (AmCare Genomics Lab, Ltd., Guangzhou, China) was conducted according to the instructions, with a 200~500 bp insert size and PE 150 bp sequencing strategy. Different coverage depths of whole genome sequencing (from 1× to 10× coverage at increments of 1×) were performed in 28 control samples. Sequencing reads were cleaned by discarding reads with a base quality less than QC20 and mapped to the reference human genome version hg19. The alignment was performed by using Burrows–Wheeler Aligner (BWA) [22].

### 2.3. Bioinformatics Analysis of AOH Detection

Quality parameters of the analysis were considered to be satisfactory with a data yield ≥ 20 Gb, Q30 > 85% and read counts ≥ 100 M. SNVs and indel variants were extracted by an in-house bioinformatics pipeline [18,23]. Based on the variantallele fraction (VAF), SNVs were classified into homozygous SNVs (B allele), heterozygous SNVs (AB allele) and nondiploid heterozygous SNVs (AAB allele and ABB allele) [24]. The parameter definitions can be seen in the Supplementary Materials. The coverage and SNVs profiles of the normal cohort at window sizes of 10 kb, 50 kb, 100 kb, 200 kb, 300 kb, and 400 kb across the human genome were also generated. The Z-score was calculated for each 200 kb window for a single sample only. The regions containing at least 10 continuous 200 kb windows with a Z-score either below minus 1 or above 1 were marked for further manual examination.

### 2.4. Validation of AOH Detection by CMA/SNP Array

According to the SNP array quality control system, DNA quality control (>250 ng, electrophoretic bands > 2000 bp), amplified and purified product quality control (the concentration of amplified purified products was more than 300 ng/μL when diluted 10 times), and fragmented product quality control (electrophoretic bands were 25–125 bp) were all achieved. Then, the labeled DNA was hybridized to the Affymetrix^®^ CytoScanTM 750K Array (Affymetrix, Santa Clara, CA, USA). After washing by the Affymetrix GeneChip^®^ Fluidics Station 450, the arrays were scanned with the GeneChip^®^ System (GCS) 3000Dx. Finally, we used the Chromosome Analysis Suite (ChAS) 14.2 software to analyze the CEL files obtained from scanning the arrays. The human reference genome was the GRCh37 (hg19) genome. The pathogenicity interpretation of CNVs and AOHs was performed according to the American College of Medical Genetics (ACMG) guidelines [25].

## 3. Results

### 3.1. Assessment of Minimum Sequencing Coverage for AOH Detection

The sequencing depth of WGS was previously shown to be positively correlated with the SNVs calling accuracy. Based on WGS data, it was estimated that SNP calling from at least 13.7× coverage depth can be >99% concordant with the genotypes obtained from CMA/SNP array, and a 15× coverage depth was recommended to have accurate single SNV genotyping [26]. However, such a high coverage depth makes it unlikely to be affordable to implement routinely.

**Figure 2:**
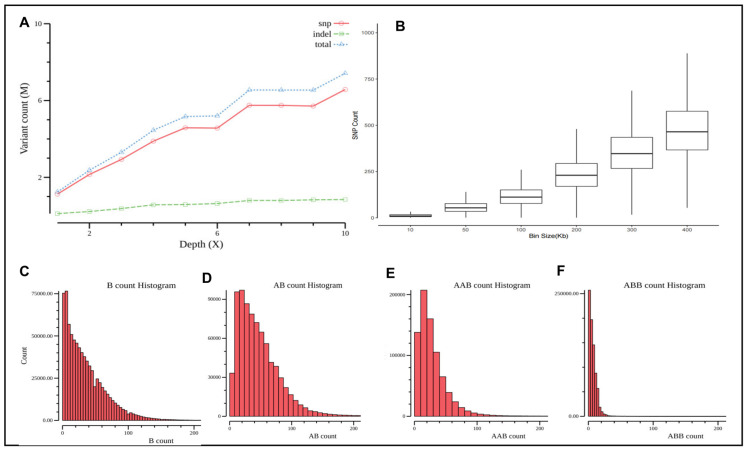
Assessment of sequencing coverage for AOH detection and the number of variant counts at variable bin size. (**A**) The number of SNVs detected at each coverage depth. (**B**) The number of B alleles, AB alleles, AAB alleles and ABB alleles at different bin size from 10 kb to 500 kb of window size. (**C**–**F**) show these four types of allele counts and distributions at a 200 kb bin size for our normal cohort.

It has previously been demonstrated that the number of called variants at 20× coverage can reach saturation at approximately 4.2 million, whereas the number of called variants at 5× can only reach 3 million, which means that approximately 30% of variants are missed [26]. Thus, we first performed WGS at a cascade of coverage depth levels of 1×, 2×, 3×, 4×, 5×, 6×, 7×, 8×, 9× and 10×. The result from Figure 2A demonstrates the number of SNVs detected at each coverage depth. It further validated that the number of call variants is positively correlated with the sequence depth and reaches a plateau at a coverage depth of nearly 7–9×.

When only reads below 5× were used for alignment, the single SNV calling accuracy declined drastically. Such low coverage coupled with random sampling alignment errors, sequencing error, etc., make higher noise than the signal, thus, making it unreliable to extract single SNV genotyping information. In sum, LP-WGS might not be suitable for AOH detection, considering the low quality of SNV, per se, and the total number of SNVs within a genomic block. A previous study also revealed a similar recommendation that at least 5× coverage appeared to be necessary for the accurate assessment of genomic variations as for the study of linkage disequilibrium [27].

To enhance the signal and increase the statistically meaningful total number of SNVs within a genomic block from 5× medium coverage data, we further extracted the number of B allele, AB allele, AAB allele and ABB allele at different bin sizes from 10 kb to 500 kb of window size. As shown in Figure 2B and Supplementary Figure S1, the total number of variants in each bin size remain in a Gaussian distribution, except for bin sizes of 10 kb and 50 kb. The number of each allele at the 100 kb bin size is relatively small, and its standard deviation is large, which makes the downstream calculation of the Z-score challenging. Considering that the AOH size of the ethnic background of the Chinese population is approximately 2 Mb in length [28,29], if we define that at least 10 continuous bin size can yield reliable AOH detection, a 200 kb bin size would be appropriate. The results of Figure 2B–F show these four types of allele counts and distributions at a 200 kb bin size for our normal cohort.

### 3.2. Performance of Detection of AOH for Prenatal Samples with CMA-seq

A total of 27 prenatal/postnatal samples (CVS, AF, CB, PB, AB) were subjected to CMA-seq and analyzed by our newly developed analytical parameters as outlined above. These 27 positive samples cover a wide spectrum of AOH types encountered during our clinical practice in recent years. Types of AOHs included chromosomal level AOHs (14.8%, 4/27), whole genome wide AOHs (3.7%, 1/27), and segmental AOHs (81.5%, 22/27). The positive findings by CMA-seq were 100% concordant with those of the CMA/SNP arrays (Table 1). This result indicated that a variety of prenatal samples were suitable for sequencing-based approaches. These data also suggested that AOHs occur ubiquitously in the human genome.

### 3.3. Evaluation and Analysis of Normal Sample and Detection of Chromosomal Level AOH

CNV and AOH analyses of 28 normal control samples verified by the CMA/SNP array were carried out as a training set by sequencing. Usually, CNV analysis of CMA/arrays requires data normalization or bias correction by mixing two sets of differently labeled genomic DNA for hybridization and signal detection [30]. Meanwhile, CNV analysis of low-pass CNV-seq takes a computationally synthesized reference from a template of approximately 10–20 batched test samples [13,20,30]. One distinct feature for CNV analysis by CMA-seq is that the data set of one single sample is used for both interchromosomal and intrachromosomal normalization, without an experimentally or a computationally generated reference. This is important for downstream continuous bioinformatics analysis of AOH detection by focusing on the sample itself.

**Figure 3:**
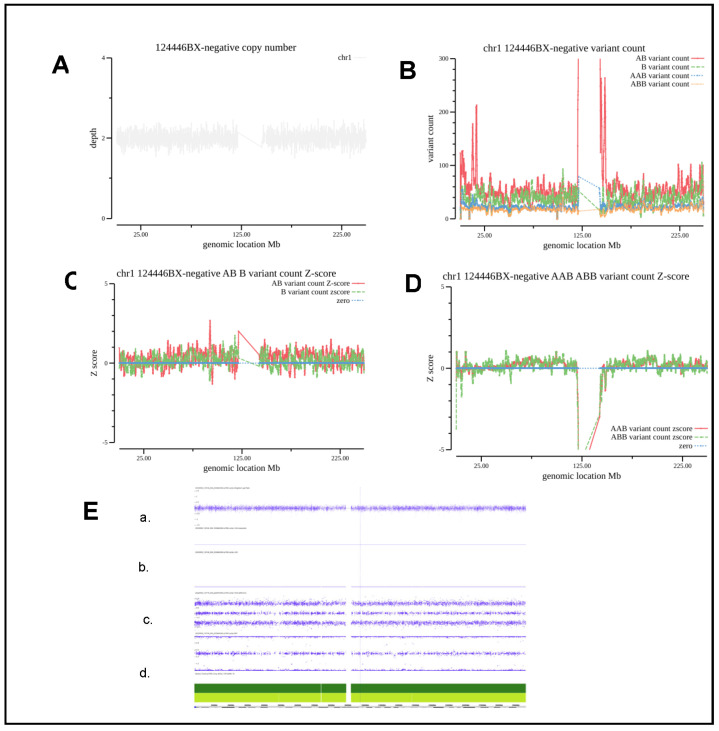
Evaluation and analysis of the CNV and AOH of a normal sample. (**A**), CNV coverage for chromosome 3 of the normal sample 124446BX. (**B**) The number of each type of allele SNVs counts is indicated as B_variant_count, AB_variant_count, AAB_variant_count and ABB_variant_count. (**C**,**D**) Z-score for each type of allele as colored by green (B_allele), red (AB_allele), orange (ABB_allele) and blue (AAB_allele). (**E**). CMA/SNP array results of a. Weighted log2 Ratio; b. Allele Difference; c. BAF; d. HG19 Coordinate.

Figure 3A shows the CNV coverage for chromosome 3 of 124446BX. The number of each type of allele SNVs counts is indicated as B_variant_count, AB_variant_count, AAB_variant_count, and ABB_variant_count in Figure 3B. The AB allele variant count was greater than the B allele variant count, which is consistent with more heterozygote variants than homozygotes for a normal individual. Meanwhile, the Z-score for each type of allele as computed to the mean value of the corresponding type of allele count across the normal control cohort at a 200 kb bin size is shown in Figure 3C,D. The Z-score for each type of allele as colored by green (B_allele), red (AB_allele), orange (ABB_allele) and blue (AAB_allele), showing that the value fluctuated around zero within the range of −1 and +1 standard deviation. There is occasionally a spike, which is probably due to a particular genomic context. When we applied the criteria that at least 10 continuous 200 kb windows with a Z-score either below −1 or above 1 candidate AOH segments, this sample was considered normal.

**Figure 4:**
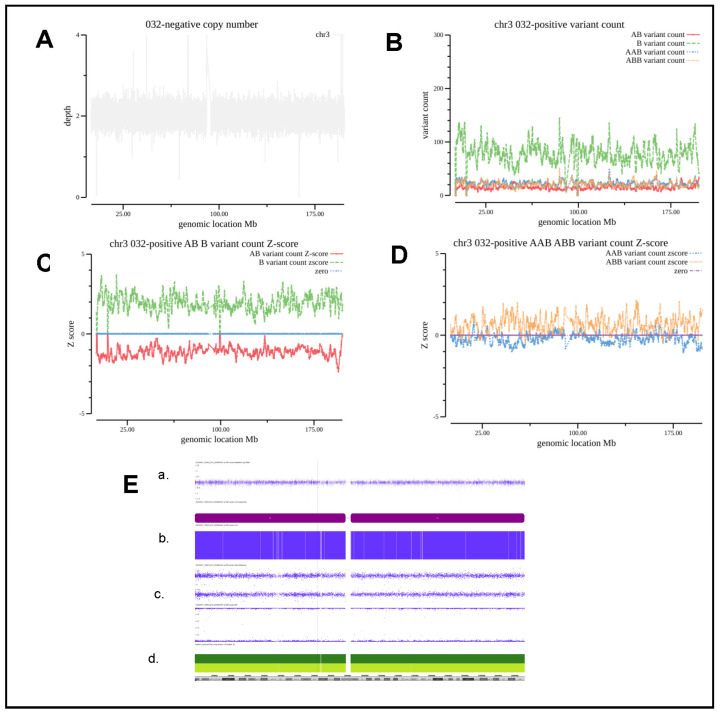
Analysis of the patient with chromosomal level AOH. (**A**), CNV coverage for chromosome 3 of Case #032. (**B**). The number of each type of allele SNVs counts as indicated. (**C**,**D**) Z-score for each type of allele as indicated. (**E**). CMA/SNP array results of a. Weighted log2 Ratio; b. Allele Difference; c. BAF; d. HG19 Coordinate.

Case #032 was from a 31-year-old pregnant woman who underwent amniocentesis for a high risk of trisomy 3 detected by noninvasive prenatal testing. Chromosome 3 encompasses at least 63 genes associated with autosomal recessive diseases, but no imprinted genes. Dandy–Walker malformation, which is a rare congenital malformation, was found by an ultrasound at 28 weeks of gestation. The parents opted to terminate the pregnancy and declined further genetic evaluation to detect potential autosomal recessive diseases. CNV of chromosome 3 in this case was normal, as shown in Figure 4A. The number of B_variant_count color in green increased dramatically; at the same time, the number of AB_variant_count also decreased, but the change in the number ABB_variant_count and AAB_variant_count is not evident in Figure 4B. When examining the Z-score distribution for B_variant_count and AB_variant_count, they show uniform values either above 1.5 or below −1.5 in Figure 4C. The divergence of the Z-score for the B and AB alleles is consistent with the genetic event of AOH occurrence, which corresponds to the BAF section of the CMA/SNP array result as indicated in Figure 4E. Genotyping error at medium coverage, suffering from insufficient depth, sequencing errors, variant call inaccuracy and statistic biases, is also significant for each SNV being called and increases the amount of noise level by interfering with single SNP-based AOH detection. However, the scheme proposed in this study for application of the total number of SNVs within a genomics block can be less affected by these genotyping errors, because the AOH event occurring at particular genomic loci belongs to a systematic event, since genotyping errors cannot influence the true occurrence of AAB and ABB alleles. As shown in Figure 4D, even with a whole chromosomal 3 AOH event, the SNVs of AAB and ABB alleles are still present at the basal level, but the Z-score distribution for the ABB_variant_count and AAB_variant_count show a small but significant shift below the zero line.

### 3.4. Evaluation and Analysis of Whole Genome Level AOH

**Figure 5:**
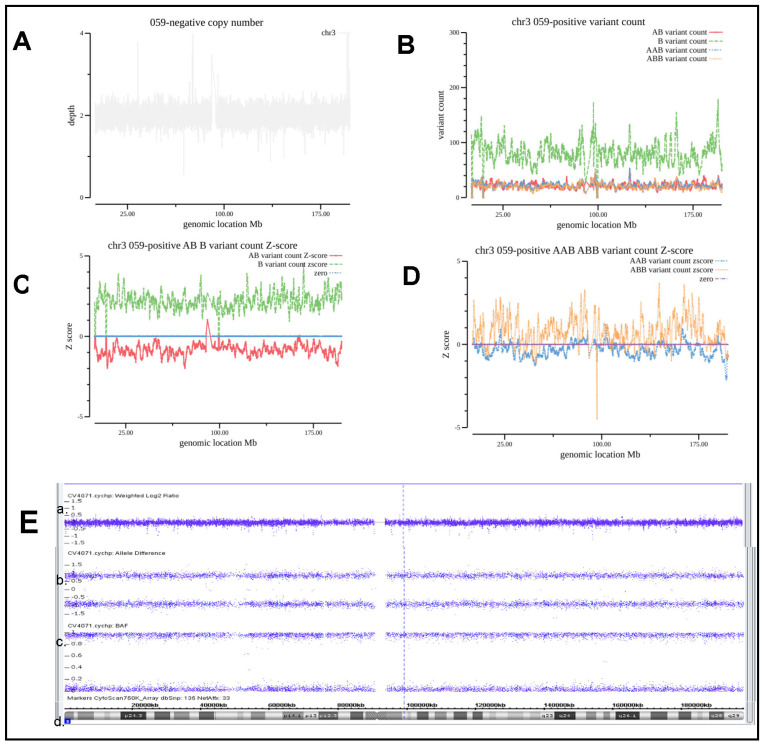
Analysis of the patient with whole genome level AOH. (**A**), CNV coverage for chromosome 3 of Case #059. (**B**) The number of each type of allele SNVs counts as indicated. (**C**,**D**) Z-score for each type of allele as indicated. (**E**) CMA/SNP array results of a. Weighted log2 Ratio; b. Allele Difference; c. BAF; d. HG19 Coordinate.

Case #059 was from a 31-year-old pregnant woman with a twin pregnancy who underwent chorionic villus sampling of the hydatidiform mole and amniocentesis of the coexisting live fetus. The CMA/SNP array testing result of the live fetus was normal, while a whole genome AOH of the hydatidiform mole was identified by the CMA/SNP array. The CNV of all the chromosomes of this hydatidiform mole was normal, as shown in Figure 5A,E of the Weighted log2 Ratio section. Figure 5B,C show that the number of B_variant_count increased and AB_variant_count decreased. The Z-score distribution and the divergence for B_variant_count and AB_variant_count meet the criteria we proposed in this study. Noticeably, as shown in Figure 5D, the Z-score distribution of AAB_variant_count remains relatively normal around the zero line, but that of ABB_variant_count shows large fluctuations below the zero line. The exact mechanism remains to be further elucidated. Paternal UPD was highly suspected in this case, but the parents declined further validation. Spontaneous abortion occurred at 23 weeks of gestation. Autopsy confirmed that the complete mole coexisted with a normal fetus.

### 3.5. Evaluation and Analysis of Segmental AOH

**Figure 6:**
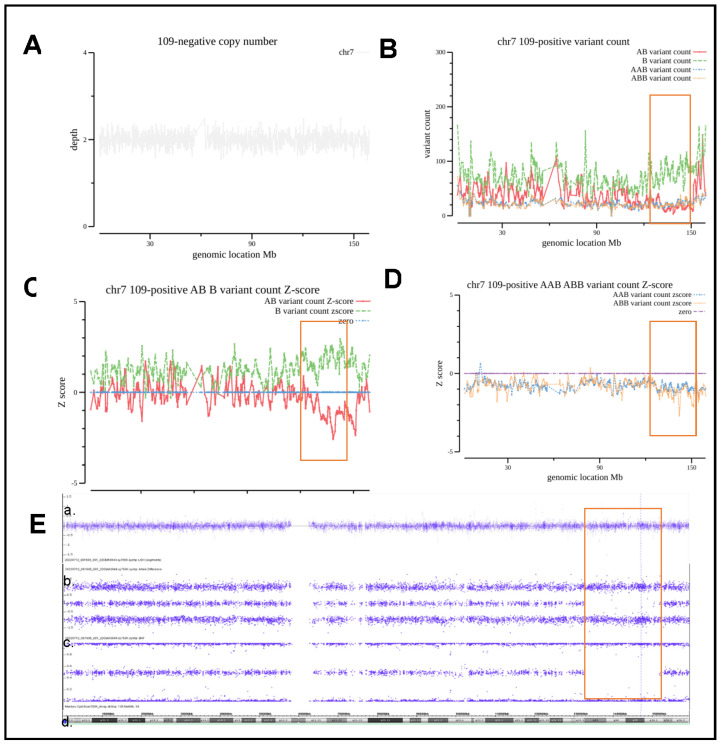
Analysis of the patient with segmental AOH. (**A**), CNV coverage for chromosome 3 of Case #109. (**B**) The number of each type of allele SNVs counts as indicated. (**C**,**D**) Z-score for each type of allele as indicated. (**E**) CMA/SNP array results of a. Weighted log2 Ratio; b. Allele Difference; c. BAF; d. HG19 Coordinate.

Case #109 is from a 39-year-old pregnant woman who underwent amniocentesis due to an advanced maternal age. The CNV of chromosome 7 in this case was normal, as shown in Figure 6A,E of the Weighted log2 Ratio section. Figure 6B,C show that the number of B_variant_count increased and AB_variant_count decreased, as well as the Z-score distribution and the divergence for B_variant_count and AB_variant_count at the genomic loci of 7q32.3q36.1 (132,300,000–151,500,000, 18.1 Mb). The other regions of chromosome 7 do not meet the criteria for AOH consideration. This region encompasses at least 15 genes associated with autosomal recessive diseases, including *AGK*, *NUP205*, *TBXAS1*, etc., but no imprinted gene. CMA testing using parental peripheral blood samples ruled out parental consanguinity and validated that fetal AOH was not UPD. The baby was delivered at 34 weeks of gestation via cesarean section due to placenta previa, and the development was normal after 1 month of follow-up. The CMA/SNP array confirmed an AOH event at q32.3q36.1 of chromosome 7 with an estimated size of 19 Mb (132,165,146–151,810,715) (Figure 6E).

For segmental AOHs, a single AOH (>10 Mb) was detected in eight samples, and multiple AOHs (2.1–109.1 Mb) were detected in 14 samples (Table 1). Chromosomes 6, 7, 14, 15 and 20 associated with imprinted genes were detected in 11 samples, five of which involved the imprinted genes of chromosome 7. Moreover, an additional 2.15 Mb–16.9 Mb AOH was detected in 8 samples by our sequencing-based method. One common phenomenon was observed through the sequencing-based approach that detected multiple short segmental AOHs more frequently than the CMA/SNP array, occasionally resulting in a discrepancy in the number of AOH segments within one genomic region. For example, in case #002, the CMA/SNP array detected one segment of AOH at 2p21p11.2 spanning 45,974,855–877,053,152 with a size of 41.1 Mb. The same sample was identified to have two AOH segments of 2p21p13.2 (45,700–72,400,000, 26.7 Mb) and 2p13.1p11.2 (75,000,000–89,000,000, 14 Mb) by CMA-seq, where heterozygous SNVs can be detected in between and verified by high read depth sequencing.

## 4. Discussion

Our study demonstrated that the identification of clinically significant AOHs by CMA-seq is in concordance with the high-density CMA/SNP array in prenatal diagnosis.

Currently, CMA is the first-tier recommendation for fetuses with ultrasound abnormalities in prenatal diagnosis, with multiple advantages over conventional karyotyping and fluorescence in situ hybridization (FISH). Next-generation sequencing (NGS) technologies have revolutionized DNA sequencing, enabling entire genomes to be sequenced more cost-efficiently [18,31,32]. In fact, limitations of CMA/SNP arrays have also gradually begun to be realized since array design preferentially covers clinically critical regions over other genomic regions, as mentioned before [33]. This uneven probe design may result in failure to detect some pathogenic CNVs [20]. In recent years, low-pass CNV-seq based on NGS has emerged as a high-resolution and cost-effective technology for genome-wide CNV detection. Several studies have shown that the resolutions of CNV-seq in detecting chromosomal microdeletions/microduplications and mosaic CNVs are very similar to those of CMA [7,18,20,31,33,34]. Compared with CMA, CNV-seq has the advantages of reduced DNA amount and quality control requirements and has been widely used in prenatal diagnosis for CNV detection [24,35,36]. However, most current low-pass WGS only focuses on CNV detection and must be combined with other methods to detect AOH in prenatal diagnosis.

AOH is a genetic characteristic known to cause human genetic diseases mainly through autosomal recessive or imprinting mechanisms. Therefore, AOH identification is strongly recommended during prenatal or postnatal genetic testing. However, most current low-pass WGS studies have only focused on CNV detection [24,36].

First, we addressed the minimal sequencing coverage threshold and genomic bin size at different read coverage depths for reliable AOH detection. For genome sequencing, the sequencing depth is variable, and increased depth could achieve higher analysis accuracy for AOH detection. In 2020, Chaubey et al. increased the detection depth in 409 clinical cases to detect AOHs and CNVs simultaneously and found that some patients with pathogenic CNVs were missed by the CMA/SNP array [21]. In 2021, Dong et al. demonstrated the feasibility of a 4× coverage for AOH analysis (≥5 Mb) and showed high consistency compared to the CMA/SNP array in 17 prenatal/postnatal cases [24].

Second, we have demonstrated the basal parameters at the particular bin size window and showed that the number of these SNV alleles remains relatively stable. Moreover, we also proposed the criteria for candidate AOH loci consideration. The number of each type of allele variant count is informative to provide the absolute number of variants within a specified genomic window block. The Z-score for each type of allele provides a numerical and sensitive measurement for the convergence and divergence of variant distribution at a very high genotyping error background. The minimal size of AOH that can be reliably evaluated currently is at least 10 continuous 200 kb windows with a Z-score either below minus one or above one. Detected AOHs are then marked for further manual examination.

Third, this study was designed to perform a medium coverage WGS method to detect AOHs in prenatal samples. By using data from 28 normal samples and 27 positive samples, the AOH by CMA-seq exhibited 100% concordance with those of CMA/SNP array analysis. In addition, CMA-seq had some additional AOH findings due to increased SNVs coverage.

Fourth, according to the ACMG recommendation, the size cutoff value of the AOH reporting region for the identification of chromosome or fragment UPD is 5 Mb [17]. In this study, the data demonstrated that the minimal AOH size reporting region for the identification of fragment UPD is 2 Mb, which can meet the ACMG reporting standard.

The limitation of this study, based on the short read sequencing method, is the inadequate capability of detecting inversions and balanced translocations, which are similar issues encountered by the CMA/SNP array approach. Thus, further algorithm optimizations and improvements are warranted with our current data. One caution of this analysis that needs to be taken into account is that the sensitivity and accuracy of the Z-score depends on the estimation on the basal size of the linkage disequilibrium block for the particular ethnic population being applied to, which would require recalibration if used elsewhere [28,29].

In conclusion, our study demonstrated that the identification of clinically significant AOHs is concordant with CMA/SNP arrays in the characterization of direct samples from prenatal genetic screenings. It is feasible to analyze the AOH by CMA-seq. This is different from previously very low coverage CNV-seq, which can only yield CNV information. Simultaneous analysis of AOHs and CNVs by CMA-seq could improve the diagnosis yield and efficiency in prenatal diagnosis. This combination ability offers an example of genomics technologies that can deliver the promise of balancing clinical testing accuracy and low economic burdens.

## Figures and Tables

**Table 1:** Summary of CMA-seq for AOH identification in 27 typical AOH positive samples, which were confirmed by the CMA/SNP array.

Samle ID	Sample Types	Clinical Indication(s)	CMA		GS		Result of Comparison	Classification	Diseases
‘001	amniotic fluid	Advanced Maternal Age, The couple had a child with chromosomal disease (46, X, iY(p10))	7q21.11q21.3(80,832,371–94,133,800)×2 hmz	13.3	7q21.11q21.3(81,000,000–94,100,000), ×2 hmz	13.1	Consistently	VUS	Silver–Russell syndrome
‘002	amniotic fluid	advanced maternal age, abnormal NIPT result (Z2 = 9.9)	2p25.3p24.3(50,814–13,311,915)×2 hmz	13	2p25.3p24.3(200,000–13,100,000), ×2 hmz	12.9	Consistently	VUS	
			2q32.3q36.3(192,341,274–230,205,775)×2 hmz	37.9	2q33.2q36.3(204,100,000–230,100,000), ×2 hmz	26	Consistently	VUS	
			2p21p11.2(45,974,855–87,053,152)×2 hmz	41.1	2p21p13.2(45,700,000–72,400,000), ×2 hmz; 2p13.1p11.2(75,000,000–89,000,000), ×2 hmz	26.7 14	Consistently	VUS	
			2q11.1q12.3(95,550,958–109,626,929)×2 hmz	14	2q11.2q12.3(98,700,000–108,200,000), ×2hmz	9.5	Consistently	VUS	
‘027	peripheral blood	parental validation for abnormal fetal CMA result. The fetus had an ultrasound abnormality of omphalocele, and the CMA indicated a 539 kb duplication on chromosome 16p11.2, which was associated with 16p11.2 duplication syndrome, as well as a 1.7 Mb duplication on chromosome Xp22.31 of unknow significance.	Xp22.31(6,455,152–8,135,568)×4		Xp22.31(6,455,152–8,135,568)x3.88		Consistently	VUS	
			4q25q28.3(111,718,067–137,498,491)×2 hmz	25	4q25q28.3(111,800,000–137,400,000), ×2hmz	25.6	Consistently	VUS	
			6q14.1q21(83,539,461–113,232,989)×2 hmz	29	6q14.1q21(83,500,000–113,200,000), ×2hmz	29.7	Consistently	VUS	transient neonatal diabetes mellitus
			7q31.1q33(112,161,869–136,999,883)×2 hmz	24	7q31.1q33(112,300,000–136,400,000), ×2hmz	24.1	Consistently	VUS	Silver–Russell syndrome
			Xp22.31p21.2(8,485,023–31,282,369)×2 hmz,	23	Xp22.31p22.2(7,900,000–10,200,000), ×2 hmz; Xp22.2(11,500,000–14,300,000), ×2 hmz; Xp22.2p22.13(15,300,000–17,800,000), ×2 hmz; Xp22.12p21.3(20,400,000–27,700,000), ×2 hmz; Xp21.3p21.2(28,500,000–31,200,000), ×2 hmz; Xp11.21(49,00,000–57,600,000), ×2 hmz; Xq21.1(80,100,000–82,400,000), ×2 hmz	2.3 2.8 2.5 7.3 2.7 2.7 2.3	Consistently	VUS	
			Xq23q25(114,567,797–128,598,791)×2 hmz	14	Xq24q25(117,900,000–128,600,000), ×2 hmz	10.7	Consistently	VUS	
					7q36.2q36.3(154,400,000–158,800,000), ×2 hmz	4.4	Additional findings	VUS	
					20p13p12.3(100,000–5,800,000), ×2 hmz	5.7	Additional findings	P	Pseudohypoparathyroidism type 1B
‘030	chorionic villus sampling	The ultrasound indicated fetal abnormality (NT = 3.3 mm)	2p12p11.2(75,632,552–87,053,152)×2 hmz,	11.4	2p12p11.2(75,600,000–87,100,000), ×2 hmz	11.5	Consistently	VUS	
			15q25.1q26.1(79,625,730–94,285,672)×2 hmz	14.7	15q25.1q26.1(78,900,000–94,200,000), ×2 hmz	15.3	Consistently	VUS	Prader Willi syndrome, Angelman syndrome
‘031	peripheral blood	systemic scleroderma	1p13.3p11.2(107,624,475_121,339,317)×2 hmz,	13.7	1p13.3p12(107,500,000–120,500,000), ×2 hmz	13	Consistently		
			1q21.1q32.2(144,077,594_207,345,376)×2 hmz,	63.3	1q21.3q22(151,200,000–155,300,000), ×2 hmz; 1q22q32.1(155,900,000–206,000,000), ×2 hmz	4.1 50.1	Consistently	VUS	
			7q11.22q21.11(71,236,774_83,356,842)×2 hmz,	12	7q11.23q21.11(75,200,000–83,400,000), ×2 hmz	8.2	Consistently	VUS	Silver–Russell syndrome
			10p14p12.1(7,588,527_29,290,352)×2 hmz	21.7	10p14p12.31(7,700,000–21,600,000), ×2 hmz; 10p12.31p12.1(22,200,000–29,600,000), ×2 hmz	13.9 7.4	Consistently	VUS	
					Xp22.33p22.31(2,100,000–8,500,000), ×2 hmz	6.4	Additional findings	VUS	
					Xp11.21(54,900,000–57,600,000), ×2 hmz	2.7	Additional findings	VUS	
					Xq13.3q21.1(74,400,000–77,000,000), ×2 hmz	2.6	Additional findings	VUS	
					Xq24q26.3(119,500,000–136,400,000), ×2 hmz	16.9	Additional findings	VUS	
					7p14.1(40,390,001–40,530,000), ×1	~140 kb, deletion	Additional findings	LP	Glutaric aciduria type III
‘032	amniotic fluid	abnormal NIPT result (Z3 = 18.49)	arr(3)×2 hmz	3UPD	chr3	3UPD	Consistently	VUS	
‘033	amniotic fluid	advanced maternal age	18q21.2q22.3(49,750,952_71,592,671)×2 hmz	21.8	18q21.2q22.3(49,900,000–71,700,000), ×2 hmz	21.8	Consistently	VUS	
‘034	peripheral blood	parental validation for fetal CMA result. The fetus had abnormal NIPT result (Z18 = −7.11) and the CMA indicated a pathogenic variant of 15Mb deletion on chromosome 18q22.1q23, as well as a 6.4 Mb duplication on chromosome 1q43q44 of unknow significance.	1q43(241,124,639_242,772,875)x1,	1.65	1q43(241,110,001–242,770,000), ×2 hmz	1.66, deletion	Consistently	P	
			2q32.3q34(192,406,884_209,298,633)×2 hmz	16.9	2q32.3q34(192,700,000–209,300,000), ×2 hmz	16.6	Consistently	VUS	
					2q31.1q31.2(176,400,000–178,600,000), ×2 hmz	2.2	Additional findings	VUS	
					18q22.1(63,100,001–63,680,000), ×2 hmz	~580 kb, deletion	Additional findings	VUS	
‘036	amniotic fluid	NT thickening	13q11q34(19,450,957–115,095,705)×2 hmz	95.6	13q11q34(19,300,000–115,100,000), ×2 hmz	95.8	Consistently	VUS	
‘048	amniotic fluid	fetal growth restriction	8q12.1q13.3(58,247,980–71,953,755)×2 hmz	13.7	8q12.1q13.3(58,400,000–73,000,000), ×2 hmz	14.6	Consistently	VUS	
‘049	amniotic fluid	nasal bone absence	3p23p21.31(31,569,962–44,915,992)×2 hmz,	13.3	3p23p22.2(31,700,000–44,400,000), ×2 hmz	13.6	Consistently	VUS	
			(21)×3	trisomy 21	chr21×3	trisomy 21	Consistently	P	Down syndrome
‘053	peripheral blood	parental validation for abnormal fetal CMA result. The pregnancy women underwent chorionic villus sampling because the first child of the couple had phenylketonuria. The CMA indicated a 208 kb deletion on chromosome 4q25 of unknown significance.	4q21.23q26(85,439,001–116,726,668)×2 hmz	31.2	4q21.23q26(85,400,000–116,500,000), ×2 hmz	31.1	Consistently	VUS	
			8q22.2q24.21(101,423,593–128,671,420)×2 hmz	27.2	8q22.3q24.21(101,700,000–129,100,000), ×2 hmz	27.4	Consistently	VUS	
			12p13.31p12.1(8,146,161–25,668,635)×2 hmz	17.5	12p13.31p12.1(8,200,000–25,700,000), ×2 hmz	17.5	Consistently	VUS	
					4q28.3q31.1(138,700,000–141,000,000), ×2 hmz	2.3	Additional findings	VUS	
					8q12.3q13.1(64,500,000–66,600,000), ×2 hmz	2.1	Additional findings	VUS	
‘054	chorionic villus sampling	twin molar pregnancy	19p13.3p11(260,911–24,462,369)×2 hmz,	19 UPD	19p13.3p12(400,000–20,900,000), ×2 hmz	20.5	Consistently	VUS	
			19q11q13.43(28,274,010–58,955,556)×2 hmz		19p12(21,900,000–24,400,000), ×2 hmz	2.5	Consistently	VUS	
					19q11q13.2(28,500,000–42,500,000), ×2 hmz	14	Consistently	VUS	
					19q13.31q13.43(43,500,000–58,900,000), ×2 hmz	15.4	Consistently	VUS	
‘055	chorionic villus sampling	omphalocele	13q12.11q13.3(22,951,566–38,289,937)×2 hmz,	15.3	13q12.11q13.3(23,100,000–38,300,000), ×2 hmz	15.2	Consistently	VUS	
			18p11.32q23(136,227–78,013,728)×3	trisomy 18	chr18×3	trisomy 18	Consistently	VUS	
‘056	peripheral blood	intrauterine demise	Xq22.1q26.3(99,176,543–135,657,966)×2 hmz,	36.4	Xq22.1q22.3(99,600,000–104,300,000), ×2 hmz; Xq23q24(111,600,000–117,100,000), ×2 hmz; Xq24q25chrX:117,400,000–123,900,000, ×2 hmz; Xq25q26.3(126,700,000–134,800,000), ×2 hmz	4.1 5.3 6.4 6.4	Consistently	VUS	
			2q21.3q24.3(135,219,538–164,635,629)×2 hmz,	29.4	2q21.2q24.1(134,700,000–158,200,000), ×2 hmz; 2q24.1q24.3(158,600,000–164,600,000), ×2 hmz	23.5 6	Consistently	VUS	
			7p22.3p21.2(50,943–14,472,953)×2 hmz,	14.4	7p22.3p21.2(500,000–14,600,000), ×2 hmz	14.1	Consistently	VUS	Silver–Russell syndrome
			12p13.33p11.21(257,935–31,063,131)×2 hmz,	30.8	12p13.33p11.21(400,000–31,300,000), ×2 hmz	30.9	Consistently	VUS	
			12q12q14.2(42,805,029–63,265,817)×2 hmz	20.4	12q12q14.2(42,600,000–64,800,000), ×2 hmz	22.2	Consistently	VUS	
					7q31.32q31.33(122,600,000–125,200,000), ×2 hmz	2.6	Additional findings	VUS	
					2p15p14(62,900,000–65,000,000), ×2 hmz	2.1	Additional findings	VUS	
					2p12p11.2(81,700,000–84,000,000), ×2 hmz	2.3	Additional findings	VUS	
‘057	Placenta	twin molar pregnancy	19p13.3p11(260,911–24,462,369)×2 hmz,	19 UPD	19p13.3p13.11(400,000–19,500,000), ×2 hmz,	19.1	Consistently	VUS	
			19q11q13.43(28,274,010–58,955,556)×2 hmz		19q11q13.43(28,500,000–59,100,000), ×2 hmz	30.6	Consistently	VUS	
‘058	amniotic fluid	congenital diaphragmatic hernia	6p25.3q27(203,877–170,896,644)×2 hmz	6 UPD	6p25.3p11.1(200,000–58,900,000), ×2 hmz	58.7	Consistently	VUS	transient neonatal diabetes mellitus
					6q11.1q27(62,100,000–171,200,000), ×2 hmz	109.1	Consistently	VUS	transient neonatal diabetes mellitus
‘059	chorionic villus sampling	twin molar pregnancy	arr(1–22,X)×2 hmz	Genome-wide	chr(1–22,X)×2 hmz		Consistently	P?	
‘061	amniotic fluid	advanced maternal age	15q22.2q25.3(61,837,131–86,568,353)×2 hmz	24.7	15q22.2q25.2(61,900,000–82,600,000), ×2 hmz	20.7	Consistently	VUS	Prader Willi syndrome, Angelman syndrome
					15q25.2q25.3(83,300,000–87,300,000), ×2 hmz	4	Consistently	VUS	Prader Willi syndrome, Angelman syndrome
					Xp22.13(18,800,001–19,180,000), x3	~380 kb, duplication	Additional findings	VUS	
‘062	peripheral blood	parental validation for fetal CMA result. The fetus had ultrasound abnormality of hyperechogenic kidneys and the CMA indicated a 1.4 Mb deletion on chromosome 17q12, which was associated with 17q12 recurrent deletion syndrome.	6q15q21(91,258,687–106,596,685)×2 hmz	15.3	6q15q21(91,400,000–106,500,000), ×2 hmz	15.1	Consistently	VUS	transient neonatal diabetes mellitus
‘064	chorionic villus sampling	megacystis, single umbilical artery	20p12.3p11.23(9,051,988–20,268,154)×2 hmz		20p12.3p11.23(9,000,000–20,300,000), ×2 hmz	11.3	Consistently	VUS	Pseudohypoparathyroidism type 1B
‘065	cord blood	amniotic fluid sample revealing karotype of 47,XXY/46,XY	arr(X)×2 hmz,(Y)×1	XXY, XUPD	arr(X)×2 hmz,(Y)×1	X UPD	Consistently	VUS	
‘066	amniotic fluid	abnormal NIPT result	3p24.1p14.2(30,639,805–63,601,962)×2 hmz,	32.9	3p24.1p21.31(30,800,000–48,200,000), ×2 hmz; 3p21.31p14.2(50,100,000–63,500,000), ×2 hmz	17.4 13.4	Consistently	VUS	
			5q11.2q13.2(54,259,794–68,826,246)×2 hmz,	14.5	5q11.2q13.2(54,100,000–71,200,000), ×2 hmz	17.1	Consistently	VUS	
			9p23p21.2(11,072,446–25,606,655)×2 hmz,	14.5	9p23p21.3(11,200,000–25,500,000), ×2 hmz	14.3	Consistently	VUS	
			18q21.1q21.32(44,375,110–57,719,351)×2 hmz	13.3	18q21.1q21.32(44,400,000–57,700,000), ×2 hmz	13.3	Consistently	VUS	
					3p26.1p25.2(6,800,000–12,300,000), ×2 hmz	5.5	Additional findings	VUS	
								VUS	
‘067	peripheral blood	parental validation for fetal CMA result. The fetus had ultrasound abnormality of congenital diaphragmatic hernia and the CMA indicated a 335 kb duplication on chromosome 7q36.1 of unknow significance.	7q36.1(151,842,964–152,178,027)×3	335 kb, duplication	7q36.1(151,840,001–152,070,000)×3	~230 kb, duplication	Consistently	VUS	
			12q23.1q24.13(98,658,599–114,095,220)×2 hmz	15.4	12q23.1q24.11(99,500,000–110,300,000), ×2 hmz	10.8	Consistently	VUS	
					16p13.3(5,910,001–6,260,000), ×1	~350 kb, deletion	Additional findings	VUS	
‘068	peripheral blood	parental validation for fetal CMA result. The fetus had ultrasound abnormality of cleft lip and palate and the CMA indicated a 14.9 Mb deletion on chromosome 18p11.32p11.21, which was associated with Chromosome 18p deletion syndrome.	arr[hg19] 9q31.1q33.3(105,122,292–129,460,914)×2 hmz,	24.3	9q31.1q33.3(104,800,000–129,400,000), ×2 hmz	24.6	Consistently	VUS	Temple syndrome, Kagami-Ogata syndrome
			12p13.2p12.1(10,121,410–24,518,044)×2 hmz	14.3	12p13.2p12.1(10,200,000–24,700,000), ×2 hmz	14.5	Consistently	VUS	
					14q24.3q31.3(77,100,000–85,800,000), ×2 hmz	8.7	Additional findings	VUS	
‘069	amniotic fluid	consanguineous marriage	9p22.3p13.1(16,315,832–38,771,831)×2 hmz	22.4	9p22.3p13.1(14,800,000–39,200,000), ×2 hmz	24.4	Consistently	VUS	
					9q21.11q21.13(71,100,000–78,500,000), ×2 hmz	7.4	Additional findings	VUS	
‘109	amniotic fluid	advanced maternal age	7q32.3q36.1(132,165,146–151,810,715)×2 hmz	19.6	7q32.3q34(132,300,000–142,000,000), ×2 hmz	9.7	Consistently	VUS	Silver–Russell syndrome
					7q34q36.1(143,100,000–151,500,000), ×2 hmz	8.4	Consistently	VUS	

## Data Availability

The datasets used and/or analysed during the current study are available from the corresponding author on reasonable request.

## Supplementary Materials

The following supporting information can be downloaded at: https://www.mdpi.com/article/10.3390/diagnostics13030560/s1, Figure S1: The number of variants dependence on the bin size selection for 24 normal samples.
