# Supplementary material for: Evaluation and Analysis of Absence of Homozygosity (AOH) Using Chromosome Analysis by Medium Coverage Whole Genome Sequencing (CMA-seq) in Prenatal Diagnosis
Source: Diagnostics (Basel). 2023 Feb 2;13(3):560. doi: 10.3390/diagnostics13030560 (PMC9914714; doi:10.3390/diagnostics13030560)
Supplement: Supplementary file 1 [file diagnostics-13-00560-s001.zip › diagnostics-2128695-supplementary.pdf]

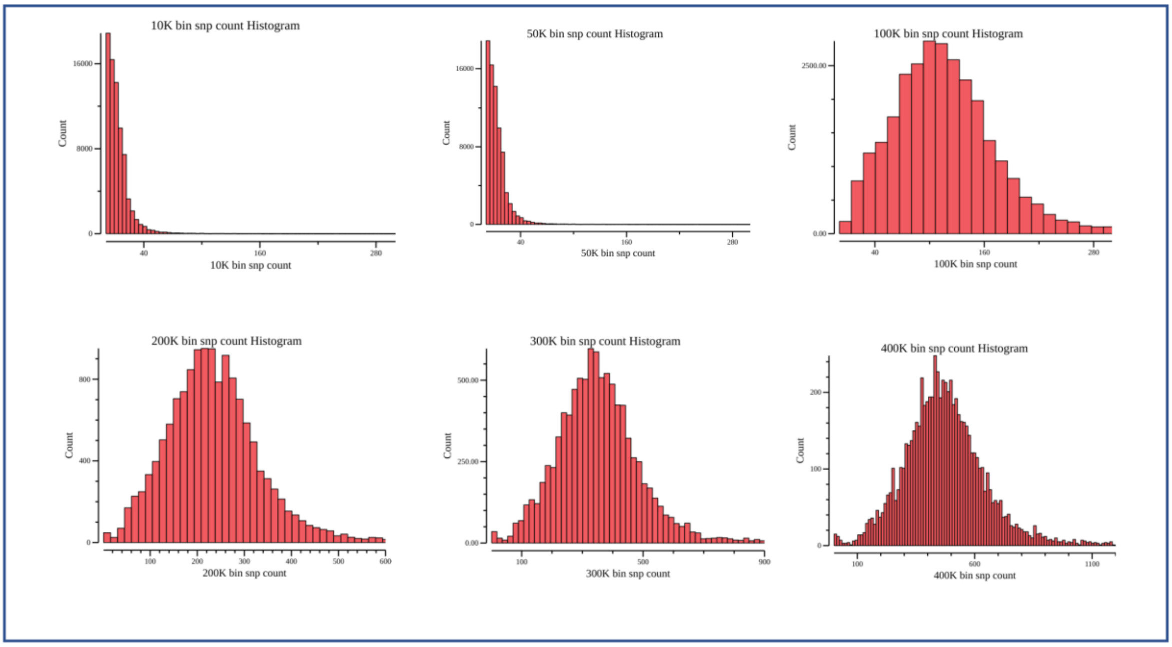

Supplemental Figure S1: The number of variants dependence on the bin size selection for 24 normal samples.
